# Expanding Arizona’s Dementia Capable System

**DOI:** 10.1093/geroni/igab046.1484

**Published:** 2021-12-17

**Authors:** David Coon

**Affiliations:** Arizona State University, Phoenix, Arizona, United States

## Abstract

Currently, 5.8 million US adults live with Alzheimer’s disease (ADRD); the number is expected to double by 2050. Arizona will experience the greatest percent increase in ADRD by 2025. This project targeted three underserved groups in order to expand Arizona’s dementia capable system: people living alone with ADRD; people with Down Syndrome or another intellectual/developmental disability (DS/IDD) aging with ADRD and their family caregivers; and people with ADRD and their caregivers in the Latino community. This presentation describes the development and delivery of the project’s educational workshops, case management services, and evidence-based programs. Over 2,220 participants have participated in workshops to date with the largest percentage being case managers, care coordinators, and discharge planners. Evaluations have been extremely positive with 86.1% being “very likely” to recommend the project to others. The presentation concludes with findings and lessons learned regarding the delivery of the project’s evidence-based programs and case management services.

